# Relationship between Serum Antibodies and *Taenia solium* Larvae Burden in Pigs Raised in Field Conditions

**DOI:** 10.1371/journal.pntd.0002192

**Published:** 2013-05-02

**Authors:** Cesar M. Gavidia, Manuela R. Verastegui, Hector H. Garcia, Teresa Lopez-Urbina, Victor C. W. Tsang, William Pan, Robert H. Gilman, Armando E. Gonzalez, the Cysticercosis Working Group in Peru

**Affiliations:** 1 School of Veterinary Medicine, Universidad Nacional Mayor de San Marcos, San Borja, Lima, Peru; 2 Department of Microbiology, School of Sciences, Universidad Peruana Cayetano Heredia, Lima, Peru; 3 Division of Parasitic Diseases, National Center for Infectious Diseases, Centers for Disease Control, Atlanta, Georgia, United States of America; 4 Duke Global Health Institute and Nicholas School of Environment, Duke University, Durham, North Carolina, United States of America; 5 Bloomberg School of Public Health, The Johns Hopkins University, Baltimore, Maryland, United States of America; 6 Cysticercosis Working Group in Peru, San Borja, Lima, Peru; Universidad Nacional Autónoma de México, Mexico

## Abstract

**Background:**

Serological tests have been used for the diagnosis of *Taenia solium* infection in pigs. However, those serological results do not necessarily correlate with the actual infection burden after performing pig necropsy. This study aimed to evaluate the Electro Immuno Transfer Blot (EITB) seropositivity with infection burden in naturally infected pigs.

**Methodology/Principal Findings:**

In an endemic area of Peru, 476 pigs were sampled. Seroprevalence was 60.5±4.5% with a statistically higher proportion of positive older pigs (>8 months) than young pigs. The logistic model showed that pigs >8 month of age were 2.5 times more likely to be EITB-positive than ≤8 months. A subset of 84 seropositive pigs were necropsied, with 45.2% (38/84) positive to 1–2 bands, 46.4% (39/84) to 3 bands, and 8.3% (7/84) to 4+ bands. 41 out of 84 positive pigs were negative to necropsy (48.8%) and 43 (51%) had one or more cysts (positive predictive value). Older pigs showed more moderate and heavy infection burdens compared to younger pigs. In general, regardless of the age of the pig, the probability of having more cysts (parasite burden) increases proportionally with the number of EITB bands.

**Conclusions/Significance:**

The probability of being necropsy-positive increased with the number of bands, and age. Therefore, the EITB is a measure of exposure rather than a test to determine the real prevalence of cysticercosis infection.

## Author Summary

*Taenia solium* is a parasite that infects humans. The parasite eggs are released into the environment with human feces in villages with inadequate sanitation. Pigs might ingest the parasite eggs and develop the larval stage named cysticercosis (cysts), mainly in the muscles and heart. If a human accidentally ingests the parasite eggs, then the cysts develop principally in the central nervous system, a serious public health problem. Serological results in pigs do not always correlate with what is observed in the necropsy; in fact, some seropositive pigs are negative or have a few cysts. This study aimed to investigate the Electroimmunotransfer Blot (EITB) with the actual pig infection burden. We selected an endemic area of Peru, and sampled 476 pigs. Seroprevalence was 60.5% and there were more old positive pigs than young ones. A subset of 84 seropositive pigs were necropsied and only 43 (51%) had one or more cysts in the whole body. Older pigs also showed more moderate and heavy infection burdens compared to younger pigs. In general, the probability of having cysts increases with the number of EITB bands. This serological assay might be a measure of exposure rather than a test to detect the real disease.

## Introduction

*Taenia solium*/cysticercosis infection is an endemic parasitic disease in less developed countries where pigs are raised as a food source [1]. The life cycle of *Taenia solium* includes the pig as the normal intermediate host, harboring the larval vesicles or cysticerci, and the human as the definitive host, harboring the adult form of the tapeworm. There are widespread economic losses due to the larval stage (cysticercosis) infection of pigs which affects the quality and safety of the pork [2]. In Mexico, for example, porcine cysticercosis caused the loss of more than half the national investment in swine production, and the losses occasioned by the destruction of meat was estimated at $43 million per year [3], [4].

Humans can also develop cysticercosis in the central nervous system (neurocysticercosis or NCC) which affects mainly older children and adults, and the economic consequences due to chronic disability are heavy [3]. In 1988, the cost was estimated in US$15 million per year only for hospital admission of new diagnosed cases of NCC in Mexico [3]. In addition, a recent study in the same country calculated a total of 25,341 (95% CR: 12,569–46,640) DALYs estimated to be lost due to the clinical manifestations of NCC [5]. In Peru, the total cost of NCC during the first 2 years of treatment (healthcare-related costs and productivity losses) was estimated in $966 per patient. This translates into 54% of a minimum wage salary during the first year of treatment and 16% during the second one. Besides, two-thirds of wage-earners lost their jobs owing to NCC and only 61% were able to re-engage in wage-earning activities [6]. However, the real costs might be underestimated since patients with calcified cysticercosis can have seizures or other neurological manifestations persisting for years even after they have been apparently effectively treated to kill cysticerci [3], [4].

The rates of porcine infection are variable but in highly endemic region over 20% to 42% of pigs may be infected [7]. Figures obtained from slaughterhouse inspection generally demonstrate lower levels of infection due to the poor sensitivity of the examination and also because infected pigs are not brought to the abattoir for slaughter since they are often confiscated without payment [2].

Infection by *T. solium* in pigs under field conditions can be diagnosed by one of three methods: necropsy, detection of cysts in the tongue, and by means of serological assays that would detect either antibodies or circulating antigens. Necropsy, or for that matter, veterinary inspection, is not particularly useful. It can only assess exposed carcass surfaces or a few exploratory incisions, and is usually by-passed as most pigs are killed clandestinely [2]. Tongue examination, although specific, is only moderately sensitive, requires highly trained personnel, is time-consuming, and entails the risk of being bitten [8] which can influence compliance. Immunological assays appear to be best suited for field surveys as pigs can be bled rapidly from the anterior cava vein and it is less dangerous for the examiner than examination of the tongue [9]. A number of assays have been developed for the detection of antibodies, including Enzyme Linked Immuno Sorbent Assay (ELISA) tests with secretory/excretory antigens [10] and fluid antigens [11], and indirect ELISA using heterologous antigens from *T. crassiceps* [12], [13] and *Cysticercus longicolis* [14].

The Electro Immuno Transfer Blot test (EITB) in combination with purified antigens [15] is highly specific and more sensitive than either ELISA or tongue examination for the detection of *T. solium* infection [8]. However, the presence of circulating antibodies against *T. solium* does not necessarily correlate with presence of cysts at necropsy, and might return positive results when necropsy is negative [16]. It is difficult, if not impossible, to distinguish antibodies found in current cyst infections from those found due to passive immunity [17] (transfer of maternal antibodies via milk), previous exposures to eggs with no established infection, or aborted infections.

However, a number of sero-surveys have shown that reaction to the seven diagnostic glycoproteins in the EITB assay does not occur randomly, but rather in distinct reaction patterns [18]. Other experiments performed mostly to study treatment alternatives have shown that some of the band pattern combinations (more than 4 bands) were more common than others in heavily infected pigs [8], [19], [20]. There have been previous studies where the cyst burden was associated with the antibody level and antigen detection. For instance, Sciutto et al (1998) found a high specificity, sensitivity and positive predictive value (PPV) in experimentally infected pigs by using an ELISA test for the detection of antibodies and antigens. However, the ELISA performance was lower in a small group of naturally infected pigs (rural conditions) for both ELISA and Immunoelectrotransference [21]. On the other hand, pigs experimentally infected with different doses of *T. solium* eggs developed a heterogeneous response; the level of serum antibodies and antigens varied with the intensity of infection, and pigs with only a few caseous cysts in muscles and/or vesicular ones in brains had no detectable antibodies [22], [23]. It has been also reported that the EITB was able to detect animals with vesicular forms of the cyst, while those with no larvae or only colloidal or caseous ones were negative to EITB in experimentally inoculated animals [24]. The present study was designed to investigate the relationship between reaction to the EITB assay and the burden of infection on examination by necropsy in naturally infected pigs from a hyperendemic area in Peru.

## Methods

### Ethics statement

This study was revised and approved by the Ethical Committee of Animal Welfare of the School of Veterinary Medicine, National University of San Marcos (Lima, Peru) which adheres to the guidelines of the Council for International Organizations of Medical Sciences (World Health Organization). Firstly, a meeting was organized with the communities to invite them to voluntarily participate in this study. An oral consent was obtained from owners (normally the Head of household) who were informed of the objective of the study as well as the minimal discomfort to the animals at the time of drawing the blood sample. Oral consent was used since most of the villagers were not able to read Spanish. After receiving permission we were allowed to work with the pigs either outside or inside the house. A registry record was created for each consenting family and their pigs. Pigs were identified by using numbered ear tags.

### General design

A serosurvey for porcine cysticercosis was conducted in 10 villages of the district of Quilcas and San Pedro de Saño, a highly endemic area in Huancayo, in the Central Peruvian Highlands. Presence of antibodies against *T. solium* was determined using EITB as previously described by Tsang et al [15]. Prevalence was estimated from the serology results with 95% confidence interval. Serology was performed within 24 hours and a subset of the EITB-positive pigs was then selected according to age and number of bands and purchased for the necropsy study. Most of the houses with positive pigs were visited, and owners were asked to sell their pigs to us. Pigs were euthanized and necropsied either at a nearby research station in Huancayo (n = 50) or transported to Lima (n = 34). The latter group was euthanized in our animal facilities at the Veterinary School, National University of San Marcos in Lima. Infection burdens were registered for every pig. An ordinal logistic regression model was fit on the necropsied pigs to assess the age, and age/band combinations that predict the infection burden in pigs. To adjust for correlated necropsy responses within households, we used Generalized Estimating Equations (GEEs) to estimate the model.

### Prevalence study

All pigs except piglets younger than 2 months of age and pregnant sows were sampled. Blood was obtained using the vacutainer system by approaching the anterior cava vein or the jugular vein. The procedure was applied with minor discomfort in the animals. The samples were centrifuged at 3000 rpm for 5 minutes and then sera was separated and frozen until tested. Pig sera were tested for cysticercosis using the EITB. The age, sex and the EITB results were registered for each of the sampled pigs.

### EITB assay

The EITB assay was performed as originally described by Tsang et al [15]. Briefly, this assay uses seven purified *T. solium* glycoprotein antigens (diagnostic bands GP50, GP42-39, GP24, GP21, GP18, GP14 and GP13, where GP stands for glycoprotein and the number refers to the molecular weight of each antigen expressed in kilo Daltons) in an immunoblot format to detect infection-specific antibodies in the serum of cases of cysticercosis in pigs. The sensitivity of the assay was claimed to be >95%, with 100% specificity initially, and reactions to one or more bands are considered positive [15].

### Necropsy evaluation

A number of EITB-positive pigs (n = 84) were purchased, euthanized and necropsied. The total number of infected pigs purchased was representative of the array of age and band pattern combinations. The price was set according to current pork market prices.

The euthanasia was performed by injecting an overdose of sodium pentobarbital (100 mg/Kg) intravenously in the pigs. In the necropsy, pigs were carefully examined for the presence of cysts in the brain and muscle tissue, including heart and tongue. Pork was carefully chopped using fine cuts (less than 0.5 cm). Cysticercosis burden was classified as follows: negative (no cysts), light if one to 10 cysts were found in the whole carcass, including the brain; moderate for 10 to 100 cysts; and heavy for those with more than 100 cysts in the whole pig. Overall a pig was considered positive if at least one cyst was found in the whole carcass.

### Data analysis

Data was analyzed using the statistical package Stata 9 (StataCorp. College Station, Texas, USA). EITB results were arbitrarily divided in four categories (negative, 1–2 bands, 3 bands, 4–7 bands). The univariate analysis was carried out by gender, location, and age. To evaluate the potential effect of maternal antibodies, pigs were classified into two groups: less or equal than 8 months, and older than 8 months. We determined the probabilities of observing different necropsy scores in pigs given different EITB levels identified, controlling for age and sex of the pig. Necropsy has 4 levels (0 to 3 categories): negative, light (1–10 cysts), moderate (11–100 cysts), and heavy infection burdens (more than 100 cysts).

An ordinal logistic regression analysis was then performed to estimate the odds ratio of being positive using the data from the prevalence study. An ordinal model was fit on the necropsied pigs since we have naturally ordered categories for necropsy results (0 through 3). The model accounted for household clustering by using Generalized Estimating Equations (GEEs) to estimate the model since we had to purchase more than one pig in some houses. Additionally, nine EITB-negative pigs were bought from the same place and were included in order to run the model to assess the probability of necropsy outcome adjusted by this category of pigs. The 95% Confidence Intervals and statistical significance were estimated, with the level of significance set at 0.05. An ordinal logistic regression model is written similarly as a logistic regression model; however, we assume that the probability distribution of falling into one of *k = 4* categories follows a multinomial distribution. Therefore, assuming (*p_i0_*,…*p_i3_*) represents the probabilities of each necropsy result for pig *i* with the cumulative probability defined as 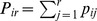 and *P_i3_* = 1, we write the ordinal logistic regression model as:
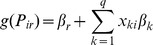
Where *r* ranges from 0 to 3, *q* represents the number of independent (predictor) variables, and *g(.)* is the cumulative logit link function.

## Results

### Animal sample characteristics

A total of 476 pigs were sampled in 10 villages from Quilcas and San Pedro de Saño Districts, in the Peruvian Central Highlands. Of those, 245 (51.5%) were females and 231 (48.5%) were males. The average age was 9.3 months (SD = 6.9). Pigs were divided in two age-groups, ≤8 months old (n = 267), and >8 months old (n = 209). The proportion of males and females for both age groups did not differ from each other statistically.

### Prevalence study

The overall seroprevalence for swine cysticercosis was calculated as 60.5% (288/476, 95% confidence interval: 55.9%–64.9%). The range of the prevalences among the ten villages varied from 38.4% in Centro, and as high as 90% in Colpar (Table 1). The proportion of EITB-positive pigs was statistically higher for older (>8 months) than younger pigs (73.2% vs 50.6% respectively, p<0.01) (Table 2). Among the seropositive pigs, the 1–2-bands category was observed in greater percentage for pigs ≤8 months old (30.7%) than older pigs (23.4%), though no statistical difference was demonstrated. However, the 3− and 4+ bands categories were statistically greater for pigs older than 8 months than young pigs (≤8 months) (3 bands: 36.8% vs 14.2%, p<0.01; 4+ bands: 12.9%vs 5.7%, p<0.05, respectively) (Table 3). Overall, it was observed that the number and proportion of seropositive pigs decreased with the increase in the number of reactive EITB bands; there was higher proportion of 1–2 EITB-band pigs (27.5%, 131/476) than 4+-EITB band pigs (8.8%, 42/476) (Table 3).

**Table 1:** Porcine cysticercosis in Quilcas and San Pedro de Saño Districts (Huancayo) by EITB.

Village	Prevalence % (pos/total)*	95% CI**
Centro	38.4 (10/26)	20.2–59.4
Canchayllo	39.4 (13/33)	22.9–57.9
Santa Cruz	40.3 (25/62)	28.1–53.6
Llacta	60.4 (32/53)	46.0–73.5
Progreso	61.2 (29/47)	46.4–75.5
27-May	64.5 (46/71)	52.5–75.8
Pampas	68.1 (47/69)	55.8–78.8
Rangra	70.4 (19/27)	49.8–86.2
Casacancha	72.1 (49/68)	59.9–82.3
Colpar	90 (18/20)	68.3–98.8
Total	60.5 (288/476)	55.9–64.9

* Pos/total = seropositive animals/total number of pigs.

** 95% CI = 95% confidence interval.

**Table 2:** EITB results by pig age from an endemic area of Central Peruvian Highlands.

Age in categories	EITB results		Total
	Negative (%)	Positive (%)	
≤8 months	132 (49.4)	135 (50.6)^a^	267
>8 months	56 (26.8)	153 (73.2)^b^	209
Total	188 (39.5)	288 (60.5)	476

a,b Proportions were statistically different (p<0.01).

**Table 3:** Distribution of EITB bands for porcine cysticercosis by two age categories.

Age in categories	Number of EITB bands				Total
	Negative	1–2	3	4+	
≤8 months	132	82	38	15	267
(%)	(49.4)	(30.7)	(14.2)^a^	(5.7)^c^	(100.0)
>8 months	56	49	77	27	209
(%)	(26.8)	(23.4)	(36.8)^b^	(12.9)^d^	(100.0)
Total	188	131	115	42	476
(%)	(39.5)	(27.5)	(24.2)	(8.8)	(100.0)

a,b Statistically different between ≤8 and >8 months old pigs at 3 EITB-bands caterogy.

c,d Statistically different between ≤8 and >8 months old pigs at 4+ EITB bands category.

The logistic regression model showed that a pig older than 8 months was 2.5 times more likely to be EITB-positive than those less or equal to 8 months after controlling for sex and village effects (OR = 2.5, 95% CI: 1.7 to 3.7). Using the village with the lowest prevalence (Centro = 38%) as the reference level, the rest of the other villages had statistically significant higher prevalences with the exception of Canchayllo, Llacta, Progreso, and Santa Cruz, after accounting for sex and age categories. For instance, Colpar showed the highest risk for porcine cysticercosis with a 15 times greater likelihood of being seropositive as compared with Centro after adjusting for age and sex (OR = 15, 95% CI: 2.9 to 83.1). Sex was not a significant risk factor for porcine cysticercosis (Table 4). The goodness of fit test for the logistic regression model was adequate (p = 0.1075).

**Table 4:** Risk factor associated with EITB-positive pigs in a Peruvian Highland (Quilcas District).

Variables		Odds ratio	95% CI
Sex	Female	Ref	–
	Male	1.0	0.7–1.6
Age in categories	≤8 months	Ref	–
	>8 months	2.5	1.7–3.7*
Villages	Centro	Ref	–
	27 Mayo	2.9	1.1–7.5*
	Canchayllo	1.2	0.4–3.4
	Casacancha	3.8	1.4–10.0*
	Colpar	15.5	2.9–83.1*
	Llacta	2.6	0.9–6.9
	Pampas	3.5	1.3–9.2*
	Progreso	2.7	0.9–7.4
	Rangra	3.8	1.2–12.3*
	Santa Cruz	1.2	0.5–3.2

* 95% CIs indicating significant difference.

### Necropsy results

A total of 84 EITB-positive pigs were purchased from the serosurveyed pigs described above, from which 34 were ≤8 months old, and 50 were >8 months. Among these pigs, 45.2% (n = 38) were positive to 1–2 bands category, 46.4% (n = 39) were positive to 3 bands only, and 8.3% (n = 7) showed 4 or more bands. Chi square computation for EITB categories and necropsy status demonstrated statistical association (p = 0.001) (Table 5).

**Table 5:** Distribution of necropsied pigs by EITB number of bands, infection burden and age^a^.

	Less or equal to 8 months				Older than 8 months				
Infection burden	EITB bands				EITB bands				Total
	1–2	3	4+	Subtotal	1–2	3	4+	Subtotal	
Negative	13	6	1	20	14	6	1	21	41
Low (1–10)	3	5	1	9	3	10	0	13	22
Moderate (11–100)	1	0	0	1	1	5	0	6	7
Heavy (100+)	2	1	1	4	1	6	3	10	14
Total	19	12	3	34	19	27	4	50	84

a Chi square for EITB categories and necropsy status showed statistical association (p<0.001).

Forty-one out of 84 EITB-positive pigs were negative to necropsy (48.8%) and 43 (51.2%) had one or more cysts, either healthy or degenerated lodged in muscular tissue (positive predictive value). Among these 43 infected pigs, 22, 7, and 14 had light, moderate and heavy infection burdens, respectively. Moderate and heavy infections were observed in the majority of older pigs (>8 months) as compared with young pigs, though no statistical difference was reached (see subtotal columns in Table 5).

The Generalized Estimating Equations (GEE) for an ordinal logistic regression model adequately fit the data according to a Score Test for the Proportional Odds Assumption (p = 0.072) accounting for household cluster. For this analysis, 9 more EITB-negative pigs were included to model the probability of necropsy outcome. EITB-negative and EITB-1 to 2 bands significantly predict the necropsy outcome compared to the reference category of EITB-4+ bands. The predicted cumulative probabilities of necropsy being observed given EITB levels can be observed in Tables 6 and 7; the probabilities are cumulative since the model is cumulative.

**Table 6:** Cumulative probabilities by necropsy levels and EITB bands in pigs >8 months old.

	EITB bands							
Necropsy	Negative		1–2		3		4+	
	Estim.*	95% CI**	Estim.	95% CI	Estim.	95% CI	Estim.	95% CI
Negative	87%	(46–98)	62%	(42–80)	29%	(17–45)	11%	(2–41)
Light	96%	(77–99)	87%	(72–95)	62%	(46–76)	32%	(9–71)
Moderate	98%	(85–99)	93%	(81–97)	75%	(60–85)	46%	(15–81)
Heavy	Ref.	–	Ref.	–	Ref.	–	Ref.	–

* Estim. = cumulative probability estimation.

** 95% CI = 95% confidence interval.

**Table 7:** Cumulative probabilities by necropsy levels and EITB bands for pigs ≤8 months old.

	EITB bands							
Necropsy	Negative		1–2		3		4+	
	Estim.*	95% CI**	Estim.	95% CI	Estim.	95% CI	Estim.	95% CI
Negative	93%	(64–99)	77%	(58–89)	46%	(25–67)	19%	(4–59)
Light	98%	(87–99)	93%	(83–97)	77%	(57–89)	49%	(16–83)
Moderate	99%	(92–99)	96%	(89–99)	86%	(69–94)	63%	(25–90)
Heavy	Ref.	–	Ref.	–	Ref.	–	Ref.	–

* Estim. = cumulative probability estimation.

** 95% CI = 95% confidence interval.

For instance, the probability of observing a light infection burden or negative pig when they are EITB-negative is 0.96 (96%) with a 95% confidence interval of 77% to 99%, significantly higher than the probability of 0.32 (32%) when they have 4 or more EITB bands with a 95% CI of 9% to 71% for pigs older than 8 months. In the same context, the probability of having light infection or being negative when a pig has 1–2 EITB-bands was 0.87 (87%) with 95% confidence interval of 72% to 95% for the same age-category pigs (Table 6).

For pigs ≤8 months, the probability of being a light infection burden or negative pig when it is EITB negative is 0.98 (98%) with a 95% confidence interval of 87% to 99%, significantly higher than the probability of 0.49 (49%) when they have 4 or more EITB bands with a 95% CI of 16% to 83%. In contrast, the probability of having light infection or being negative when a pig has 1–2 EITB-bands was 0.93 (93%) with 95% confidence interval of 83% to 97% for the same age-category pigs (Table 7).

From those computations, we calculated the predicted probabilities, not the cumulative ones, for pigs to have cysticercosis according to the number of bands (Table 8). For example, for an EITB-negative-≤8 months old pig, the probability of being necropsy-negative is 93% while the probability of being heavily infected is only 1%. In the same context, a ≤8-months old-pig with 4 or more EITB bands has a probability of 36.8% of being classified as heavily infected (more than 100 cysts). The probability of being heavily infected increases up to 53.9% for a >8 month-old pig when it reacts to 4 or more EITB bands. In general, regardless of the age of the pig, the probability of having more cysts (parasite burden) increases proportionally with the number of EITB bands (Table 8), while the probability of being necropsy negative reduces as EITB bands increases.

**Table 8:** Predicted probabilities of necropsy status by EITB-number of bands and pig age-categories.

NECROPSY STATUS	EITB bands in pigs ≤8 months old				EITB bands in pigs >8 months old			
	Neg	1–2	3	4+	Neg	1–2	3	4+
Negative (no cysts)	93.3	77.3	45.5	19.4	87.3	62.9	29.4	10.7
Light (1–10 cysts)	5.0	15.8	31.4	29.6	9.2	24.2	33.0	21.6
Moderate (11–100 cysts)	0.8	2.9	8.7	14.3	1.5	5.3	12.4	13.8
Heavy (more 100 cysts)	1.0	3.9	14.3	36.8	2.0	7.6	25.2	53.9

## Discussion

We evaluated the performance of the EITB test to diagnose actual swine cysticercosis in pigs from rural communities where the disease has been declared hyperendemic (Central Peruvian Highlands). The meat inspection and the tongue examination to detect the parasite are not practical and almost impossible to occur in the study. The main limitation of veterinary inspection, an insensitive procedure, is that pigs with low cysticercercal burden might not be detected; therefore, lightly infected carcasses would remain in the chain food and the parasite transmission would persist in the population [21]. Thus, immunological assays appeared to be best suited for this type of field surveys. Pigs were bled rapidly from the jugular or anterior cava vein and sera was then analyzed using the EITB test. Among the serological tests available worldwide, EITB has become one of the most common one to study human and pig cysticercosis in endemic countries such as Peru.

Prior to the development of the EITB, serological diagnosis of porcine cysticercosis was hampered by the lack of a reliable test to establish previous exposure to *T. solium* eggs. However, the presence of *T. solium* specific antibodies detected by EITB, or any diagnostic test for that matter, does not always correlate with the detection of parasites at necropsy; in fact, some serologically positive pigs have had subsequent negative necropsy results [10], [21], [23], [25]. The interpretation of serological tests may vary not only with the tests (e.g. EITB, ELISA) but also with the type of infection. For instance, it has been shown that the ELISA specificity was very high for the detection of both antibodies and antigens in pigs from commercial farms; this test was, furthermore, found to be highly sensitive and specific in experimentally infected pigs. However, the sensitivity, specificity and PPV did not even reach 50% for ELISA-antigen detection and was very low for antibody detection when evaluating pigs under rural conditions [21].

The initial sensitivity and specificity of the EITB was initially claimed to be >95%, and 100% [8], [15]. Although we were not able to estimate sensitivity and specificity in this study, we found a low PPV of 51.2% even though the study area was a hyperendemic for swine cysticercosis. A positive serological result in the face of a negative necropsy could occur because of the transfer of maternal antibodies to the offspring which remain for several months, prior effective treatment, past infection that has been cleared (degenerated or caseous cysts), exposure to *T. solium* eggs without development of observable cysts (not enough time for the cyst to develop at the time of the necropsy), or the ingestion of non-viable or infective eggs among other possible explanations. In addition, it is described the effect of “secondary transmission” where seropositive pigs (1–2 EITB bands) were negative at necropsy while a 3-EITB-band pig had very few degenerated and healthy cysts in the whole carcass [26]. Similar low PPV has been reported from pigs maintained under rural conditions in Mexico [21]. These findings may confirm a disadvantage of the EITB test, particularly in pigs from rural areas and natural exposure. It may be interesting to consider the EITB test as an indicator of environmental contamination by detecting seropositive pigs rather than only a tool to assess actual infection status.

There is another aspect related with the EITB result interpretation. We would like to point out that the comparison of the EITB test between different research groups may not be appropriate since the technique and its standardization are more complicated than other serological tests (e.g. ELISA). Different antigen sources and antigen preparation might, additionally, influence the final results of the EITB.

In this study, we are reporting that there is an increasing trend of being necropsy positive with the number of EITB bands regardless the age of the pigs. Interestingly, the predictive probabilities of having cysts were higher in pigs that reacting to more diagnostic bands (3 and 4+) than pigs having a few bands (1–2 bands). The EITB tests was moderate in assessing the heavy infection burden for pig cysticercosis; that probability was observed in older pigs (>8 months of age) with 4+ EITB bands (53.9%) while the same infection status had a probability of 25.2 when pigs reacted to 3 bands. Thus, there are two important factors in order to estimate the actual infection status; first is the pig age, the older ones have higher probability of being infected. Secondly, the number of EITB bands; the probability of having viable cysts (active infection) increases directly with the number of reacting EITB bands. Similar observation was also described by other researchers, where the number of EITB bands increased with the infective dose and it was directly correlated with the duration of the infection in experimentally infected pigs [27].

Although the presence of antibodies in necropsy negative pigs may, in some ways, limit the use of EITB, these results suggest that diagnostic patterns did not happen at random and that these results are related to the final infection outcome. In fact, we have shown a higher proportion of seropositives in older (>8 months old) than younger (≤8 months old) pigs (73.2% vs 50.6%, respectively). A study in Mozambique also demonstrated that the prevalence of swine cysticercosis increased with the age of the animals [28]. Similarly, Garcia et al [29] reported that pig cysticercosis prevalence increased with age. Older pigs might have had more chance to get exposure to *T. solium* eggs than younger ones, and more time for cyst to develop and trigger the production of circulating antibodies. Older pigs were more than 2 times likely to be seropositive as compared with younger ones. Besides, it could be possible that younger pigs are protected during their first months of life against parasite infection, perhaps due to the presence of maternal cysticercus-antibodies (passive immunity) and become susceptible later after the slow clearance of those antibodies. Passively transferred antibodies for cysticercosis are detected by EITB several months after piglets are born from naturally infected sows [17].

Knowing the true infection status of a pig in an endemic area is crucial for the implementation of a control program. Seropositive pigs might not be necessarily confiscated if reacting with one or two EITB bands, while pigs reacting to more EITB bands might be either eliminated, treated, or may help to identify *T. solium* hotspot areas. As demonstrated for humans, the seroprevalence defined by the presence of three or more positive EITB bands increased 13% each time distance to the nearest tapeworm carrier halved [30]. Monitoring seroprevalence of pigs and identifying those reacting to more EITB bands (cyst carriers) might help to locate tapeworm carriers (humans) and hotspots in the environment.

Pig cysticercosis seroprevalence is hyperendemic in the Central Highlands of Peru. In this study eight out of the ten villages had more than 50% of their pigs seropositive to cysticercosis, likely one of the highest rates as compared with other endemic countries. For instance, the apparent prevalence of cysticercosis for antibodies or antigen detection ranged from 10–35% in Mozambique [31], and from 24.6–32.2% in Cameroon [32]. However, those serological high rates may represent either disease (presence of cysts) or only exposure to *T. solium* eggs with no disease at all but detection of circulating antibodies. Although the 10 villages were alike in terms of socioeconomic, educational level and demographic characteristics, there were villages with significant higher risk to have EITB-positive pigs as compared with Centro (the lowest prevalence). There might be other related factors that we did not evaluate at the time of this study (e.g. latrine presence, nearby tapeworm carriers, etc). Serological results, therefore, need careful interpretation since there are often more antibody-positive pigs than pigs harboring cysts, thus the positive predictive value of EITB could be low [33]

This experiment also suggests that the burden of infection was aggregated. The final burden of infection in an infected pig is related to the infective dose and to a series of events that happen before, during and after the infection challenge. Usually, the population distributions of helminthes indicate a tendency towards aggregation, meaning that the majority of parasites are harbored by a minority of host [34]. Aggregation is generally recognized as an important factor in the dynamics of host-macroparasite interactions, and it has been found to be relevant in stabilizing population dynamics in a coexisting equilibrium [34], [35]. Aggregation tends to influence the interactions that regulate parasite numbers, such that the interactions influence a larger proportion of the parasite population. The impact of macroparasites upon host populations is critically dependent upon parasite frequency distributions [36] (Gonzalez, personal communication).

We demonstrated that EITB serology for the diagnosis of cysticercosis in pigs has to be interpreted carefully. Pigs reacting to 4 or more EITB bands have higher probabilities to be infected, harboring more cysts, than pigs with less than 4 bands. In the same context, younger pigs showed less probability of having cysts than older pigs (≥8 months old). There is a proportional trend between the number of EITB bands and the cyst burden; the greater number of bands the higher probability of harboring cysts in the muscles. Transient cysticercosis-antibodies might be the result of passive immunity in piglets, unsuccessful *T. solium* egg infection (e.g. immunity of the host, ingestion of non-viable eggs), and antiparasitic treatment in pigs. Further studies might be necessary to standardize or refine the current serological test to increase its sensitivity in detecting the true disease (cyst presence).

Other Members of the Cysticercosis Working Group in Peru are Silvia Rodriguez, Luis Gomez, Guillermo Lescano, Viterbo Ayvar, Hermes Escalante, Juan Jimenez and Guillermo Gonzalvez
